# Medical associations’ guidance on caring for patients experiencing incarceration in the United States

**DOI:** 10.1371/journal.pone.0330361

**Published:** 2025-09-03

**Authors:** Nicholas V. Nguyen, Tyler A. Nguyen, Kathleen M. Akgün, Amen Sergew, Matthew F. Griffith, Erin S. DeMartino

**Affiliations:** 1 Biomedical Ethics Research Program, Mayo Clinic, Rochester, Minnesota, United States of America; 2 Alix School of Medicine, Mayo Clinic, Rochester, Minnesota, United States of America; 3 Yale School of Medicine, Yale University, New Haven, Connecticut, United States of America; 4 VA-Connecticut Healthcare System, West Haven, Connecticut, United States of America; 5 Independent Researcher, Denver, Colorado, United States of America; 6 Pulmonary Sciences and Critical Care Medicine, University of Colorado School of Medicine, Aurora, Colorado, United States of America; 7 VA Eastern Colorado Health Care System, Aurora, Colorado, United States of America; 8 Division of Pulmonary and Critical Care Medicine, Mayo Clinic, Rochester, Minnesota, United States of America; Kore University of Enna: Universita degli Studi di Enna 'Kore', ITALY

## Abstract

The United States has one of the highest incarceration rates in the world. Carceral status can complicate clinical encounters in community and academic settings for an already medically vulnerable population. While it is likely physicians will encounter patients experiencing incarceration in their practice, there are few educational opportunities dedicated to ensuring delivery of healthcare that protects patient dignity, autonomy, and privacy. Professional medical associations can play a role in filling this physician knowledge gap. The goal of this analysis is to catalogue and analyze the current landscape of official medical association documents addressing healthcare of people experiencing incarceration. A systematic Internet search was conducted of American Medical Association House of Delegates associations and their existing documents. Out of 116 associations included in the systematic search, 16 groups published materials on incarceration. From these 16 associations, 44 documents were identified and coded thematically. Documents served four main purposes: education on incarceration (28/44), clinical guidance (25/44), logistical guidance (27/44), and policy advocacy (30/44). Common topics included medical conditions of people experiencing incarceration, patient factors antecedent to incarceration, and specialty or population-specific information. Few medical associations have published material on incarceration and the paucity of pragmatic clinical guidance was particularly pronounced. A lack of resources from medical associations can lead to variability and lapses in best healthcare practices when treating patients experiencing incarceration. Medical associations should consider developing guidance for clinicians to maximize this patient population’s autonomy and dignity.

## Introduction

The United States (U.S.) has one of the highest incarceration rates worldwide, with more than 2 million people experiencing incarceration (individuals detained in federal, state, and municipal correctional facilities) [1,2]. People who identify as Black, Hispanic, and Indigenous are overrepresented in jail and prison populations [3–6]. People who are incarcerated are more likely to present for medical care with traumatic injuries, infectious diseases, and chronic conditions than their community-dwelling counterparts, reflecting the complex impact of incarceration on health [7–16]. Importantly, people who are incarcerated have the right to receive healthcare, based on the Eighth Amendment to the U.S. Constitution and its prohibition against cruel and unusual punishment, yet the enforcement of this right is variable[17,18].

Often, medical needs can be met within correctional facilities, but when they cannot, people experiencing incarceration must leave the correctional facility to receive care in neighboring clinics and hospitals. The frequency with which community and academic clinicians provide care to incarcerated patients [19,20] is only expected to rise, given the U.S.’s high incarceration rate and the burden of chronic illness among people experiencing incarceration [12,13]. In these settings, patients experiencing incarceration may encounter clinicians who have received little to no training about carceral health [21,22], harbor biases [23,24], and misunderstand what is and is not legally and ethically permissible[25]. Healthcare providers can have knowledge deficits in scenarios such as shackling [26], patient privacy and correctional officer presence [27], and surrogate decision-making [28]. Physicians and other healthcare providers have reported feeling inadequately prepared in caring for patients experiencing incarceration and have departed from standard, patient-centered practice patterns during the treatment of this population [19,20,25,29].

To avoid perpetuating health disparities, healthcare providers should have access to resources that orient them to special considerations when caring for incarcerated individuals, such as their potential medical complexity and social disenfranchisement. Professional medical associations can be one such source.

Professional medical associations are hubs for medical specialties and subspecialties. They help establish and maintain a professional identity for individual members and the specialty at large [30], and facilitate networking and professional development for early career members [30,31]. As importantly, they serve as resources for continuing education after postgraduate training, by disseminating advances in clinical practices and setting standards of care and professional ethical standards [32]. Medical associations are uniquely situated to address the knowledge gap on carceral healthcare—the healthcare of patients experiencing incarceration—among non-correctional clinicians. Caring for this vulnerable population requires special consideration, especially when interacting with law-enforcement and navigating differing policies of correctional systems and healthcare facilities [33].

Incarceration and its associated health disparities have recently garnered increased attention, particularly in the context of the COVID-19 pandemic, excessive force by police, and policy measures to address mass incarceration [34]. The confounding effects of a high incarceration rate, increased burden of chronic and acute diseases, and an aging prison population result in community-practicing clinicians likely encountering these patients [35]. Yet, they may be unfamiliar with how to provide care that respects patients' rights and dignity in these circumstances. To our knowledge, a thoroughcharacterization of education and guidance published by medical associations on carceral healthcare has not previously been published. Therefore, we sought to systematically identify and analyze official publications from medical associations regarding the health care of people experiencing incarceration, to understand the deficits that can be addressed to better serve this vulnerable population.

## Methods

### Data source: Identifying relevant documents

This study was a systematic scoping review and content analysis of published healthcare professional materials on carceral health. To conduct a systematic search for relevant documents, a member of the study team (N.V.N) first identified a list of 125 professional medical associations that are members of the American Medical Association (AMA) House of Delegates. The AMA House of Delegates is the legislative and policy making body of the AMA; a list of affiliated medical associations is found on the AMA website [36]. An initial search of materials allowed for a review of associations spanning from large (e.g., American College of Physicians) to small (e.g., the American College of Mohs Surgery). As such, prior to the systematic search, two study team members (N.V.N and E.S.D.) reviewed the list of medical associations and excluded those that were unlikely to have any contact with patients who are detained in prisons and jails (e.g., International Society of Hair Restoration Surgery). This complete list of exclusions is reported in S1 Table.

Between July 11, 2023 and August 19, 2023, we reviewed the first 20 results of an Internet search engine (Google), combining the association’s name and a standard list of search terms, which included, “*prison*,” “*incarceration*,” “*carceral*,” “*correctional*,” “*jail*,” and “*detainee*.” If a document was not identified through the search, we repeated the same search process using the association website’s search query, and subsequently inserted the search terms into PubMed. The research team searched for any educational information, clinical guidance, or any other material on carceral healthcare that was endorsed by the medical association. Presence of the medical association’s name in the title or depiction of its seal on the document were considered indicators of official endorsement. If an association updated a document, the original publication date was noted but the most recent version of the document was included for analysis. Because these data were publicly available and there was no involvement of human subjects, institutional review board approval was not required.

### Categorization of documents

Two study team members (N.V.N and T.A.N) independently reviewed the associations’ documents, seeking to categorize them by purpose: educational, clinical guidance, logistical guidance, or policy.

We developed inclusion criteria for each category. Sometimes, the document’s purpose was evident from its title (e.g., an “official policy statement” or “clinical guidelines”). When it was not, the two study team members reviewed the contents and deliberated until consensus. The senior author (E.S.D.) participated in deliberation if consensus was not reached.

Descriptive data points, such as publication date, publication dates of initial and revised documents, and authorship (e.g., singular authors, committees) were also recorded during this categorization.

### Thematic analysis

We conducted a thematic analysis of the documents. After in-depth reading of 5 documents, two authors (N.V.N and E.S.D) inductively developed an initial codebook of themes addressed [37]. Non-mutually exclusive themes addressed included living conditions in jails and prisons, disparities in incarceration experienced by minoritized racial and ethnic groups, COVID-19 and specific sub-populations such as women, youth, transgender people.

Next, two study team members (N.V.N and T.A.N) independently performed a thematic analysis of all included documents in parallel. The codebook and definitions were refined iteratively during analysis. The final coding analytic framework was applied to all identified documents that were publicly available as of August 19, 2023. REDCap, an electronic data collection and management software, was used to manage these data. The team met weekly to generate consensus; all documents were coded by both analysts. Coding uncertainty was addressed through thorough team deliberation with the senior author (E.S.D.) until consensus was reached. Once the thematic analysis was completed, descriptive statistics were calculated using Excel.

## Results

**Fig 1:**
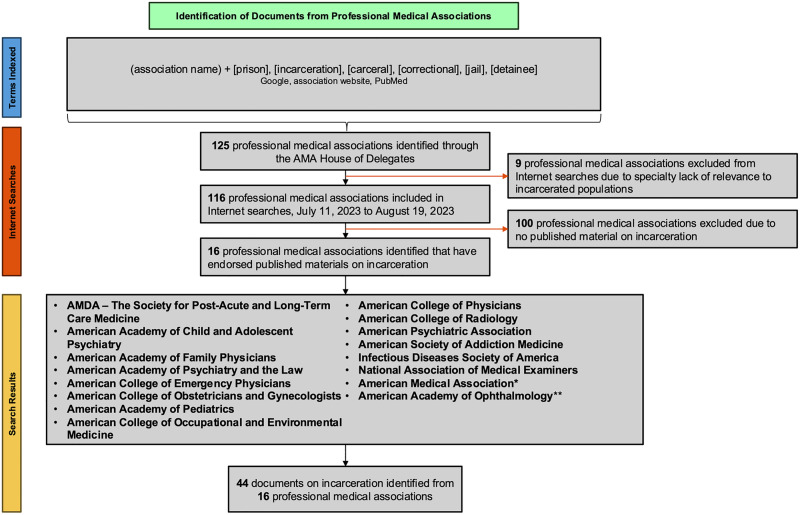
Identifying professional medical association documents addressing incarceration. Internet search for documents on healthcare for individuals who are incarcerated was conducted; the AMA House of Delegates list of specialty professional medical association was used as a reference list; 9 associations were excluded due to specialty irrelevance to correctional healthcare (e.g., American Society of Cytopathology, Undersea and Hyperbaric Medical Society, Aerospace Medical Association); a total of 16 associations published endorsed material on carceral healthcare. (*One statement was published by the AMA Opioid Task Force, a coalition of associations organized by the AMA; **One statement was published by the American Academy of Ophthalmology presenting an overview of how to bill for the care of patients experiencing incarceration, but providing no clinical guidance).

Among the 125 specialty and subspecialty associations in the AMA House of Delegates, 9 were excluded from the Internet searches due to a specialty’s lack of contact with carceral healthcare (See S1 Table). Of the 116 associations included in the search, 16 (13.8%) published at least one document regarding carceral health (See Fig 1).

We identified 44 unique documents (S2 Table), with a median of 2 statements per association (range 1–11). The American Psychiatric Association promulgated the greatest number, 11 documents. Notably, the American Academy of Dermatology (AAD) website published a magazine article by dermatologists with experience caring for individuals who are incarcerated, containing information about mass incarceration and clinical pearls; because it did not meet specified criteria for an official association document, it was excluded from our final analysis [38].

**Fig 2:**
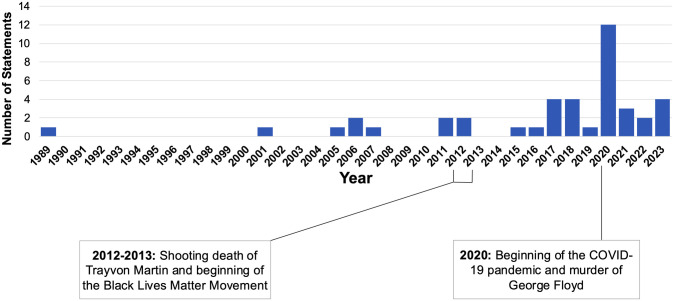
Distribution and frequency of published documents from 1989 to 2023. The number of documents published per year were plotted on a timeline with major historical events related to racial inequality and the COVID-19 pandemic. Two documents were excluded from this temporal analysis (publication dates not found).

Most documents (32/44, 72.7%) were published between 2015−2023. There were several documents published in the years around 2012−2013, coinciding with the Black Lives Matter movement and national attention to racial injustice in the wake of Trayvon Martin’s murder. Notably, there was a sharp increase in publication by medical associations in 2020, which marked the beginning of the COVID-19 pandemic and the growth of the Black Lives Matter Movement after George Floyd’s murder (See Fig 2).

### Document categories frequencies

We found that documents appeared to be written with four main objectives: 1) educational materials for healthcare providers, 2) clinical guidance, 3) logistical guidance, and 4) policy advocacy. Several documents accomplished multiple objectives.

Of the 44 documents, 28 (63.6%) contained educational content on incarceration, carceral healthcare, or the health status of people experiencing incarceration. For example, the American College of Obstetricians and Gynecologists (ACOG) provided information on the reproductive needs of people who are incarcerated.

*The majority of incarcerated women are parents and are of reproductive age, which has important implications for their reproductive health care needs. Adolescents in these settings also have reproductive health care needs that often are neglected. Additionally, the rapid turnover of incarcerated individuals and unpredictable timing of jail and detention releases can present challenges for health care delivery and continuity of care.* – Reproductive Health Care for Incarcerated Pregnant, Postpartum, and Nonpregnant Individuals Committee Opinion Number 830 [39].

Twenty-five of the 44 (56.8%) documents contained tailored clinical guidance and recommendations for caring for individuals detained in correctional facilities. These recommendations included taking precautions to protect a patient’s privacy and ensuring that people who are incarcerated receive treatment matching the standard of care within that particular field. For example, the American College of Emergency Physicians (ACEP) included practical guidance about interacting with patients in custody in the emergency department.

*Care provided or needed must be unbiased and must not be influenced by officers. If safety is an issue, allow the officer to be in clear view. The healthcare provider should not jeopardize his or her own safety. The shackles and restraints may or may not need to be removed.* - Recognizing the Needs of Incarcerated Patients in the Emergency Department [40].

Twenty-seven of the 44 documents (61.4%) included administrative, operational or organizational recommendations for interacting with the carceral system, which we labeled logistical guidance. For instance, the American Academy of Ophthalmology published a document with detailed instructions for seeking reimbursement after caring for patients detained in prisons and jails [41]. Logistical guidance documents also included suggestions for ensuring continuity of care after hospital discharge and navigating the correctional staff hierarchy to identify with a person authorized to make decisions as illustrated by the American Academy of Psychiatry and the Law.

*Psychiatrists navigating in the correctional environment need to successfully communicate and interact with staff who operate in a structured chain of command. This chain includes a hierarchy, from line officers to supervising officers, with progressive ranks up to the facility warden or chief administrator.* - The American Academy of Psychiatry and the Law Practice Resource for Prescribing in Corrections [42].

Lastly, 30/44 (68.2%) documents highlighted policies, either by advocating for policy change or by simply sharing information on policies, laws, or statutes relevant to the practice of that specialty, or to the welfare of people in prisons and jails more broadly. These included model language for policies at the level of a healthcare institution or recommending members promote policy change at the local, state, or national level. The following example is from the American Academy of Family Physicians (AAFP):

*Due to increased incarceration time for many individuals, the AAFP calls for a review and changes to the cash bail system, as it increases the risk of both short- and long-term negative health outcomes, exacerbates socioeconomic disparities, and is racially biased against individuals who are Black, Indigenous, and people of color.* - Incarceration and Health: A Family Medicine Perspective (Position Paper) [43].

Among advocacy-oriented policy statements, we further characterized their focus. Some documents addressed multiple topics. Among these: advancing or changing aspects of healthcare for people detained in prisons and jails (26/44; 59.1%), broader calls for reforming the criminal-legal system (17/44; 38.6%), and specific calls for improving correctional living conditions (13/44; 29.5%). S3 Table contains example excerpts from policies addressing these themes.

### Common themes

Our thematic analysis of the associations’ official documents revealed several throughlines, irrespective of the document’s intended purpose. These content areas included general overviews of carceral healthcare, characterization of patients experiencing incarceration as medically vulnerable, addressing ethical quandaries that arise in the care of patients who are incarcerated, and highlighting certain subpopulations, such as youths and women. Table 1 presents overall prevalence of themes and Table 2 provides selected excerpts of all content areas that were extrapolated from this analysis.

**Table 1:** Specific content areas and special populations.

Category	n (total number of documents)
**Common Themes**	
Minoritized Racial and Ethnic Incarceration Disparities	15
Antecedent and Socioeconomic Factors	10
Acute and Chronic Medical Conditions	17
Substance Use Disorders	13
Behavioral Health Disorders	28
Right to Healthcare	16
Immigrant Detention	11
Special Populations	21
COVID-19	10
Ethical Concerns	20
Specialty-specific Content	30
**Special Populations**	
Women, Pregnancy Care	14
Youth	14
Older Adults	7
Sexual Minorities	3
Transgender People	7

**Table 2:** Example excerpts from medical association documents by theme.

Theme	Medical Association	Title	Quote
**Theme**			
Racial and Ethnic Incarceration Disparities	**American Psychiatric Association**	**The Impact of COVID-19 on Incarcerated Persons with Mental Illness**	Prior to the COVID-19 pandemic, persons with mental illness (PwMI), and people of color with mental illness in particular, were disproportionately represented in the jail and prison system.
Antecedent and Socioeconomic Factors	**American Academy of Child & Adolescent Psychiatry**	**Policy Statement on the Jurisdiction of the Juvenile Court System**	One third of incarcerated youth have lived through five or more adverse childhood events (ACEs), which are widely understood to be a source of toxic stress with negative impacts on development, behavior, and health.
Acute and/or Chronic Medical Conditions	**American College of Physicians**	**Health Care During Incarceration: A Policy Position Paper from the American College of Physicians**	Incarcerated populations have a higher burden of certain chronic health conditions compared with the noninstitutionalized U.S. population. In a Bureau of Justice Statistics 2011–2012 survey, state and federal prisoners were about 1.5 times more likely to report ever having high blood pressure, diabetes, or asthma.
Substance Use Disorders	**American Society of Addiction Medicine**	**Public Policy Statement on Access to Medications for Addiction Treatment for Persons Under Community Correctional Control**	Rates of substance use disorders among those on probation or parole are significantly and consistently higher than those of the general population, and the risk of opioid overdose is considerably higher for persons on probation or parole than for the general population.
Behavioral Health Disorders	**American College of Emergency Physicians**	**Recognizing the Needs of Incarcerated Patients in the Emergency Department**	[The] incarcerated often receive substandard care at correctional facilities and face a unique set of personal and environmental challenges not least of which are high suicidal tendencies and mental health issues.
Right to Healthcare	**American Academy of Psychiatry and the Law**	**The American Academy of Psychiatry and the Law Practice Resource for Prescribing in Corrections**	Besides the professional duty of psychiatrists and other mental health providers to relieve suffering, the treatment of incarcerated persons with mental illness, including pretrial detainees, is guaranteed by the Constitution through Estelle v. Gamble (1976) and its progeny.
Immigrant Detention	**American Academy of Pediatrics**	**Detention of Immigrant Children**	Studies of detained immigrants, primarily from abroad, have found negative physical and emotional symptoms among detained children, and posttraumatic symptoms do not always disappear at the time of release.
COVID-19	**American Psychiatric Association**	**Position Statement on Growing Fear over Coronavirus Spread and Mental Health Impact in ICE Detention Centers**	The American Psychiatric Association urges the Department of Homeland Security in conjunction with ICE to consider supervised release of detainees or to find an alternative for those detainees charged with an immigration violation. Detaining children and adults during the coronavirus pandemic gravely threatens their mental and physical health and endangers their lives.
Ethical Concerns	**American College of Obstetrics and Gynecology**	**Health Care for Incarcerated Women**	[The American College of Obstetrics and Gynecology,] ACOG, supports policies restricting the use of shackling on incarcerated people throughout pregnancy, labor and delivery, transport, and postpartum recovery.
Specialty Specific	**Infectious Diseases Society of America**	**HCV Guidance Updates Recommendations for Screening and Treating Key Populations**	Chronically infected people in prisons — and those whose jail sentences are sufficiently long enough to complete a total course of antiviral therapy — should receive antiviral therapy according to [the American Association for the Study of Liver Diseases/Infectious Diseases Society of America,] AASLD/IDSA[,] guidance while incarcerated.
**Special Populations**			
Women	**American College of Obstetrics and Gynecology**	**Reproductive Health Care for Incarcerated Pregnant, Postpartum, and Nonpregnant Individuals**	Rates of sexually transmitted infections are disproportionately higher among incarcerated women, as compared with incarcerated men and nonincarcerated women. In one study, up to 11% of incarcerated women were diagnosed with chlamydia and 3% with gonorrhea. In 2015, 1.3% of women in state and federal prisons were known to be infected with HIV (human immunodeficiency virus).
Youth	**American Academy of Pediatrics**	**Advocacy and Collaborative Health Care for Justice-Involved Youth**	Facilities should provide youth who are confined for more than 1 week comprehensive preventive services, including a comprehensive history and physical examination; mental health and substance use screening; dental screening.
Older Adults	**AMDA – The Society for Post-Acute and Long-Term Care Medicine**	**Addressing an Expected Increase in Long Term Care Continuum Residents with Criminal/Correctional Histories**	THEREFORE BE IT RESOLVED, that AMDA – Dedicated to Long Term Care Medicine (AMDA) Board explore the projected needs of the growing aging criminal justices system’s inmates (…) in the long-term care continuum (LTCC) relating to this subject including: 1. How to safely manage an expected influx of residents with a criminal history in LTCC settings.
Sexual Minorities	**American College of Physicians**	**Health Care During Incarceration: A Policy Position Paper from the American College of Physicians**	Position 16: [The American College of Physicians,] ACP recommends that policymakers and administrators ensure all incarcerated persons who identify as lesbian, gay, bisexual, transgender, queer, and others (LGBTQ+) be treated with dignity and respect in a correctional environment that is safe, nondiscriminatory, and gender-affirming.
Transgender People	**American Academy of Child and Adolescent Psychiatry**	**Transgender Youth in Juvenile Justice and other Correctional Systems**	Consistent with this position, the Academy recommends that (…) facilities should take the necessary precautions to ensure the safety of every youth in their custody, including transgender youth.

Many documents presented overviews of social, economic, and health factors that disproportionately impact incarcerated populations. The American Psychiatric Association called attention to the overrepresentation of minoritized racial and ethnic groups in correctional facilities, stating, “Black Americans are imprisoned at a rate of approximately five times the rate of White Americans, while Hispanic Americans are incarcerated at 1.3 times the rate of White Americans [44].” Another frequently mentioned topic was that people who are incarcerated have a Constitutional right to healthcare. The American Academy of Psychiatry and the Law document presented the legal framework underpinning this right in a practice resource for psychiatrists [42], emphasizing that their carceral status should not interfere with receiving healthcare.

We also discovered documents that included themes that educate clinicians on circumstances arising from their patients’ conditions of confinement. Ten documents included content about the impact of the COVID-19 pandemic in correctional facilities, with some acknowledging the enhanced risk of aggregate living conditions and emphasizing that delays in routine care should be avoided, in spite of the pandemic [45,46]. Other considerations specific to patients’ confinement included ethical concerns in the care of patients who are detained (e.g., shackling) and the incarceration of refugees and migrants. Additionally, many associations separately discussed at-risk subpopulations, including women, youth, older adults, sexual minorities, and transgender people. These documents acknowledged that disparities in numerous marginalized groups are often amplified in carceral settings.

## Discussion

### Scarcity of Education and Guidance

We discovered that only 16 out of the 116 specialty association members of the AMA House of Delegates included in the search have published material on the care of individuals who are incarcerated. Topics spanned from educational attainment to socioeconomic status to chronic and acute medical conditions and to substance use and behavioral health disorders. We found that these statements help to establish a foundation for clinicians to understand overarching social and health disparities prevalent in populations involved in the criminal-legal system.

Only 12 out of 116 associations included in the search promulgated documents that contained pragmatic guidance to improve or manage the clinical care of patients experiencing incarceration, with guidance specific to their respective specialties. A scant number of documents offer guidance on delivering healthcare that affirms the human dignity of a patient who is incarcerated or, on a more basic level, how clinicians should interact with correctional staff in a clinical setting. Clinicians already struggle to address surrogate decision-making, in-hospital shackling, and correctional officer hospital presence as they care for patients who are incarcerated [25,28,47,48]. Because few educational or pragmatic guidance documents exist, clinicians are left to their own devices to navigate these often-complex dynamics.

### Power of professional medical associations

We found that a small number of associations have published policy documents that advocate for the improvement of the criminal-legal system, correctional living conditions and healthcare of individuals experiencing incarceration. Yet, several associations have taken a significant stance on providing humanity-affirming care. Notably, the American College of Obstetricians and Gynecologists (ACOG) has published on key topics, including opposition to universal shackling, providing clinical guidance for clinicians to treat pregnant and post-partum patients in correctional settings, calls for lawmakers to protect and enhance the quality of healthcare for people who are incarcerated, and advocacy that has catalyzed federal legislative protections for patients who are incarcerated during childbirth [39,49,50].

Medical associations have historically advocated on behalf of marginalized populations. For example, the AMA has issued numerous policy statements in support of healthcare for sexual minorities and transgender patients [51–53]. The AMA, American Academy of Neurology, and the American Psychiatric Association have published position and policy statements advocating for equitable healthcare for patients with physical and intellectual disabilities [54–56]. Likewise, the American Academy of Pediatrics has published clinical guidance on the care coordination of pediatric patients with intellectual disabilities [57]. Medical associations could play a pivotal role in filling this void for patients experiencing incarceration, as they have with other disenfranchised patient groups.

Professional medical associations have also long influenced health practice and policy in the U.S. For example, the AMA secured a Center for Medicare and Medicaid rule to minimize prior authorization decisions, and has strongly advocated for affordable, over-the-counter naloxone [58,59]. Official documents issued by medical associations reflect the values and mission of the organization and can create a standard for members to follow. Furthermore, policy position statements can influence the creation of policy at the organizational (e.g., association, hospital system), local, state, or even federal levels.

Medical associations can influence treatment of individuals involved with the criminal legal system in both positive and negative ways, even outside of clinical settings, as illustrated by the story of “excited delirium.” “Excited delirium” has been listed as a cause of death in multiple police-involved fatalities, despite a lack of consensus on diagnostic criteria or clear pathophysiologic explanation for how delirium caused death. “Excited delirium” was formally endorsed by the American College of Emergency Physicians (ACEP) in 2009, in a document entitled, “White Paper Report on Excited Delirium Syndrome” [60]. It has since been shown to have been disproportionately diagnosed among people from minoritized racial and ethnic groups who are forcibly subdued by law enforcement [61]. In 2020 and 2021, respectively, the American Psychiatry Association (APA) and the AMA both formally opposed the diagnosis [62,63]. ACEP revisited the issue in 2021 by reporting recent research on the condition and in 2023, voted to officially derecognize “excited delirium” as a diagnosis, citing the term’s social harms [64,65].

Given that only 16 medical associations have attempted to advance dignified healthcare for people in jails and prisons, all medical associations should examine and acknowledge their potential impact on the health of people experiencing incarceration, either through action (published documents, advocacy) or inaction. Furthermore, they can catalyze a call to action for members to deliver compassionate medical care, policymakers to create policies that respect the dignity and rights of people experiencing incarceration, and correctional systems to collaborate with non-correctional healthcare workers to improve the health and healthcare of individuals involved in the criminal-legal system [66].

### What’s Next?

While physicians and other health professionals are ethically obligated to provide care to patients experiencing incarceration that mirrors the patient-centered care they deliver to all patients, they often lack knowledge requisite to protect this patient populations’ rights and interests. Physicians recognize these training deficits and desire additional resources, acknowledging that these knowledge gaps are negatively impacting the care of an already disenfranchised population [67].

An important way to enhance clinician knowledge on carceral healthcare beyond medical association statements is through partnerships between academic medical centers and correctional facilities to provide learners with hands-on experience and knowledge of best practices. Several medical schools have collaborated with state and local correctional systems to develop such experiences, such as the University of Connecticut and University of Massachusetts Medical School [68]; yet, there are very few programs across the U.S. that facilitate this partnership [68]. Moreover, these programs only target clinicians-in-training and not practicing clinicians, failing to address education needs within the current clinician workforce.

Professional medical associations can empower clinicians to provide outstanding care to patients experiencing incarceration, standardize clinical practice guidelines, and even influence correctional and health policy. From our analysis, most documents were published after 2012, reflecting the medical community’s growing attention to mass incarceration over the past decade. Medical associations published 12 documents in 2020 alone, perhaps responding to racial disparities in COVID-19 deaths and the increased media spotlight on police violence and mass incarceration during the expansion of the Black Lives Matter Movement. This increase in attention may have influenced medical associations to promulgate statements and guidance to confront social injustices. However, medical associations should publish proactively rather than reactively. Furthermore, only 13.8% of AMA House of Delegates member organizations included in this analysis have issued guidance on this often-overlooked population. The remaining medical associations should consider developing materials about incarceration and health, narrowing the knowledge gap for healthcare providers. In turn, clinician-leaders can be better-informed as they provide bedside care or advocate for practice or policy change on behalf of future patients.

### Limitations and future directions

Our study has several limitations. We relied on publicly available statements accessible through the association websites and through PubMed. Additional relevant documents could also be available only to medical association members, or may not be indexed by the search engines in this study. Statements varied in level of detail from brief, broad statements to nuanced, specialty-specific content. In addition, we were unable to evaluate whether healthcare professionals are referencing existing guidance and, when it is present, how often they influence clinical care. Future efforts should include needs assessments with clinicians from specialties commonly interfacing with patients who are incarcerated to gauge their knowledge gaps, so that societies can provide helpful and specific resources to educate and empower their respective workforces.

## Conclusion

People experiencing incarceration face significant challenges in their health and healthcare; yet, very few professional medical associations in the U.S. offer members guidance on their unique care needs. Our findings highlight unmet educational opportunities for clinicians treating patients experiencing incarceration outside of jails and prisons. This can lead to community and academic clinicians providing inconsistent and variable healthcare to this patient population nationwide, which may cause ethical harms or violations. Specialized knowledge and accommodation are needed to uphold this patient population’s constitutionally-afforded right to receive community-standard healthcare. As trusted and prominent ambassadors in science and medicine, professional medical associations should consider how their obligations to train and educate clinicians translate to the care of people detained in correctional facilities, and how their actions stand to influence broad, societal debates around mass incarceration and equity.

## Supporting information

S1 Table Excluded Medical Associations from Analysis. This table lists the nine associations excluded from the systematic Internet searches due to specialty irrelevance to carceral healthcare.
(DOCX)

S2 Table Documents Identified by Association. Forty-four documents were identified through systematic Internet searches. This table catalogs them by association and summarizes the primary focus of each association’s collection of documents. URLs are provided where available; if a document is no longer publicly accessible, no URL is listed.
(DOCX)

S3 Table Focus of Advocacy-Oriented Policy Statements. This table presents example excerpts illustrating each advocacy-oriented theme.
(DOCX)

## References

[1] Carson EA, Kluckow R. Correctional Populations in the United States, 2021 – Statistical Tables: US Department of Justice, Office of Justice Programs. 2023. https://bjs.ojp.gov/document/cpus21st.pdf

[2] Widra E, Herring T. States of Incarceration: The Global Context. Prison Policy Initiative. 2021.

[3] Mauer M, King RS. Uneven justice: State rates of incarceration by race and ethnicity: The Sentencing Project; 2007 [09/27/2023]. https://www.issuelab.org/resources/695/695.pdf

[4] Subramanian R, Riley C, Mai C. Divided Justice: Trends in Black and White Jail Incarceration, 1990-2013: Vera Institute of Justice; 2018 [09/27/2023]. https://www.vera.org/downloads/publications/Divided-Justice-full-report.pdf

[5] Wildeman C, Wang EA. Mass incarceration, public health, and widening inequality in the USA. Lancet. 2017;389(10077):1464–74. doi: 10.1016/S0140-6736(17)30259-3 28402828

[6] Carson EA. Prisoners in 2021: Bureau of Justice Statistics; 2022 [June 1, 2023]. https://bjs.ojp.gov/sites/g/files/xyckuh236/files/media/document/p21st.pdf

[7] Haber LA, Erickson HP, Ranji SR, Ortiz GM, Pratt LA. Acute Care for Patients Who Are Incarcerated: A Review. JAMA Intern Med. 2019;179(11):1561–7. doi: 10.1001/jamainternmed.2019.3881 31524937

[8] Hammett TM. HIV/AIDS and other infectious diseases among correctional inmates: transmission, burden, and an appropriate response. Am J Public Health. 2006;96(6):974–8. doi: 10.2105/AJPH.2005.066993 16449578 PMC1470637

[9] Spaulding AC, Seals RM, Page MJ, Brzozowski AK, Rhodes W, Hammett TM. HIV/AIDS among inmates of and releasees from US correctional facilities, 2006: declining share of epidemic but persistent public health opportunity. PLoS One. 2009;4(11):e7558. doi: 10.1371/journal.pone.0007558 19907649 PMC2771281

[10] Dolan K, Wirtz AL, Moazen B, Ndeffo-Mbah M, Galvani A, Kinner SA, et al. Global burden of HIV, viral hepatitis, and tuberculosis in prisoners and detainees. Lancet. 2016;388(10049):1089–102. doi: 10.1016/S0140-6736(16)30466-4 27427453

[11] Wilper AP, Woolhandler S, Boyd JW, Lasser KE, McCormick D, Bor DH, et al. The health and health care of US prisoners: results of a nationwide survey. Am J Public Health. 2009;99(4):666–72. doi: 10.2105/AJPH.2008.144279 19150898 PMC2661478

[12] Maruschak LM, Berzofsky M, Unangst J. Medical problems of state and federal prisoners and jail inmates, 2011-12: Bureau of Justice Statistics; 2015 [09/27/2023]. https://bjs.ojp.gov/content/pub/pdf/mpsfpji1112.pdf

[13] Binswanger IA, Krueger PM, Steiner JF. Prevalence of chronic medical conditions among jail and prison inmates in the USA compared with the general population. J Epidemiol Community Health. 2009;63(11):912–9. doi: 10.1136/jech.2009.090662 19648129

[14] Leddy MA, Schulkin J, Power ML. Consequences of high incarceration rate and high obesity prevalence on the prison system. J Correct Health Care. 2009;15(4):318–27. doi: 10.1177/1078345809340426 19633334

[15] Greene M, Ahalt C, Stijacic-Cenzer I, Metzger L, Williams B. Older adults in jail: high rates and early onset of geriatric conditions. Health Justice. 2018;6(1):3. doi: 10.1186/s40352-018-0062-9 29455436 PMC5816733

[16] Saloner B, Parish K, Ward JA, DiLaura G, Dolovich S. COVID-19 Cases and Deaths in Federal and State Prisons. JAMA. 2020;324(6):602–3. doi: 10.1001/jama.2020.12528 32639537 PMC7344796

[17] Estelle v. Gamble. US: Supreme Court. 1976. p. 97.

[18] Alsan M, Yang CS, Jolin JR, Tu L, Rich JD. Health Care in U.S. Correctional Facilities - A Limited and Threatened Constitutional Right. N Engl J Med. 2023;388(9):847–52. doi: 10.1056/NEJMms2211252 36856624

[19] Barnett H, Taylor Z, Booker L, Ricklefs C, Veltri K, Ervin DV, et al. Training Among Noncustodial Health Care Workers Caring for Patients Experiencing Incarceration: A Preliminary Investigation. J Correct Health Care. 2023;29(6):411–20. doi: 10.1089/jchc.22.11.0087 37917880

[20] Douglas AD, Zaidi MY, Maatman TK, Choi JN, Meagher AD. Caring for Incarcerated Patients: Can it Ever be Equal?. J Surg Educ. 2021;78(6):e154–60. doi: 10.1016/j.jsurg.2021.06.009 34284945

[21] Simon L, Tobey M. A National Survey of Medical School Curricula on Criminal Justice and Health. J Correct Health Care. 2019;25(1):37–44. doi: 10.1177/1078345818820109 30602333

[22] Kraus ML, Isaacson JH, Kahn R, Mundt MP, Manwell LB. Medical Education About the Care of Addicted Incarcerated Persons: A National Survey of Residency Programs. Subst Abus. 2001;22(2):97–104. doi: 10.1080/08897070109511449 12466672

[23] Redmond N, Aminawung JA, Morse DS, Zaller N, Shavit S, Wang EA. Perceived Discrimination Based on Criminal Record in Healthcare Settings and Self-Reported Health Status among Formerly Incarcerated Individuals. J Urban Health. 2020;97(1):105–11. doi: 10.1007/s11524-019-00382-0 31628588 PMC7010870

[24] Hashmi AH, Bennett AM, Tajuddin NN, Hester RJ, Glenn JE. Qualitative exploration of the medical learner’s journey into correctional health care at an academic medical center and its implications for medical education. Adv Health Sci Educ Theory Pract. 2021;26(2):489–511. doi: 10.1007/s10459-020-09997-4 33074443 PMC8041700

[25] Brooks KC, Makam AN, Haber LA. Caring for Hospitalized Incarcerated Patients: Physician and Nurse Experience. J Gen Intern Med. 2022;37(2):485–7. doi: 10.1007/s11606-020-06510-w 33409890 PMC7787594

[26] Haber LA, Pratt LA, Erickson HP, Williams BA. Shackling in the Hospital. J Gen Intern Med. 2022;37(5):1258–60. doi: 10.1007/s11606-021-07222-5 35091917 PMC8971251

[27] Griffith MF, O’Brien JK, Sergew A, Wynia MK, Carno M, Akgün KM. Profiling, Privacy, and Protection: Ethical Guidance When Police Are Present at Bedside. Ann Am Thorac Soc. 2022;19(6):890–4. doi: 10.1513/AnnalsATS.202111-1245PS 35081329

[28] Larsen E, Drabiak K. Medical decision-making when the patient is a prisoner. Clinical Ethics. 2022;18(2):142–7. doi: 10.1177/14777509221133660

[29] Batbold S, Duke JD, Riggan KA, DeMartino ES. Decision-Making for Hospitalized Incarcerated Patients Lacking Decisional Capacity. JAMA Intern Med. 2024;184(1):28–35. doi: 10.1001/jamainternmed.2023.5794 38048093 PMC10696514

[30] Vail EA, Nadig NR, Sahetya SK, Vande Vusse LK, Walkey AJ, Liu V, et al. The Role of Professional Organizations in Fostering the Early Career Development of Academic Intensivists. Ann Am Thorac Soc. 2020;17(4):412–8. doi: 10.1513/AnnalsATS.201908-573PS 31800295 PMC8174059

[31] Liang PS, Andres SF, Perumpail RB, Shah R, Strauss AT, Pointer S. The Importance of Professional Societies as Academic Homes. Clin Gastroenterol Hepatol. 2023;21(10):2450–6. doi: 10.1016/j.cgh.2023.05.005 37301221

[32] Kinney ED. The origins and promise of medical standards of care*. Virtual Mentor. 2004;6(12):virtualmentor.2004.6.12.mhst1-0412. doi: 10.1001/virtualmentor.2004.6.12.mhst1-0412 23260291

[33] Nguyen NV, Riggan KA, Eber GB, Williams BA, DeMartino ES. A Primer on Carceral Health for Clinicians: Care Delivery, Regulatory Oversight, Legal and Ethical Considerations, and Clinician Responsibilities. Mayo Clin Proc. 2025;100(2):292–303. doi: 10.1016/j.mayocp.2024.09.009 39797865 PMC11950980

[34] LeMasters K, Brinkley-Rubinstein L, Maner M, Peterson M, Nowotny K, Bailey Z. Carceral epidemiology: Mass incarceration and structural racism during the COVID-19 pandemic. Lancet Public Health. 2022;7(3):e287–90. doi: 10.1016/S2468-2667(22)00005-6 35247354 PMC8890762

[35] Skarupski KA, Gross A, Schrack JA, Deal JA, Eber GB. The Health of America’s Aging Prison Population. Epidemiol Rev. 2018;40(1):157–65. doi: 10.1093/epirev/mxx020 29584869 PMC5982810

[36] Member organizations of the AMA House of Delegates: American Medical Association; [1/19/24]. https://www.ama-assn.org/house-delegates/hod-organization/member-organizations-ama-house-delegates

[37] Clarke V, Braun V. Teaching thematic analysis: Overcoming challenges and developing strategies for effective learning. The Psychologist. 2013;26(2).

[38] Margosian E. Caring For Incarcerated Individuals: DermWorld; 2022 [July 24, 2023]. https://digitaleditions.walsworth.com/publication/?i=748286&article_id=4276230&view=articleBrowser

[39] Reproductive Health Care for Incarcerated Pregnant, Postpartum, and Nonpregnant Individuals: The American College of Obstetricians and Gynecologists; 2021 [01/17/2024]. https://www.acog.org/-/media/project/acog/acogorg/clinical/files/committee-opinion/articles/2021/07/reproductive-health-care-for-incarcerated-pregnant-postpartum-and-nonpregnant-individuals.pdf

[40] Recognizing the Needs of Incarcerated Patients in the Emergency Department: American College of Emergency Physicians; 2006 [2/26/2024]. https://www.acep.org/administration/resources/recognizing-the-needs-of-incarcerated-patients-in-the-emergency-department

[41] How to Bill for Patients Seen While Incarcerated: American Academy of Ophthalmology; 2018 [July 18, 2025]. https://www.aao.org/practice-management/news-detail/how-to-bill-patients-seen-while-incarcerated

[42] AAPL Practice Resource for Prescribing in Corrections: American Academy of Psychiatry and the Law; 2018 [2/24/2024]. https://www.psychiatry.org/File%20Library/Psychiatrists/Practice/Clinical%20Practice%20Guidelines/AAPL-Corrections-Resource-Document.pdf 29884616

[43] Incarceration and Health: A Family Medicine Perspective (Position Paper): American Academy of Family Physicians; 2022 [2/24/2024]. https://www.aafp.org/about/policies/all/incarceration.html

[44] Position Statement on Engaging Law Enforcement Personnel and Correctional Staff to Address Mental Health and Racial Inequities in Jails and Prisons: American Psychiatric Association; 2023 [January 18, 2025]. https://www.psychiatry.org/getattachment/f130cbd5-82e9-449a-8631-3bcee9123894/Position-Inequities-Jails-Prisons.pdf

[45] Caring for Patients During the COVID-19 Pandemic: Managing Justice Involved People with Addiction During COVID-19 Pandemic: The American Society of Addiction Medicine. https://downloads.asam.org/sitefinity-production-blobs/docs/default-source/guidelines/covid-19/6-tf_managing-justice-involved-persons-with-addiction-during-the-covid-19-pandemic_final.pdf?sfvrsn=69ba58c2_2

[46] Strengthening the Response to COVID-19 in Correctional Facilities: Infectious Diseases Society of America; 2020 [January 18, 2025]. https://www.idsociety.org/globalassets/idsa/public-health/covid-19/covid-19-in-correctional-facilities.pdf

[47] Haber LA, Pratt LA, Erickson HP, Williams BA. Shackling in the Hospital. J Gen Intern Med. 2022;37(5):1258–60. doi: 10.1007/s11606-021-07222-5 35091917 PMC8971251

[48] Griffith MF, O’Brien JK, Sergew A, Wynia MK, Carno M, Akgün KM. Profiling, Privacy, and Protection: Ethical Guidance When Police Are Present at Bedside. Ann Am Thorac Soc. 2022;19(6):890–4. doi: 10.1513/AnnalsATS.202111-1245PS 35081329

[49] Health Care for Incarcerated Women: The American College of Obstetricians and Gynecologists; [01/17/2024]. https://www.acog.org/advocacy/policy-priorities/health-care-for-incarcerated-women

[50] The First Step Act, Stat. 132 Stat. 2018. p. 5194.

[51] Access to Basic Human Services for Transgender Individuals H-65.964: American Medical Association; 2017 [July 18, 2025]. https://policysearch.ama-assn.org/policyfinder/detail/transgender?uri=%2FAMADoc%2FHOD.xml-H-65.964.xml

[52] Committee opinion no. 695: Sterilization of women: Ethical issues and considerations. Obstetrics & Gynecology. 2017;129(4). 10.1097/AOG.0000000000002023 28333823

[53] Health Care Needs of Lesbian, Gay, Bisexual, Transgender and Queer Populations H-160.991: American Medical Association; 2024 [July 18, 2025]. https://policysearch.ama-assn.org/policyfinder/detail/conversion%20therapy?uri=%2FAMADoc%2FHOD.xml-0-805.xml

[54] Aravamuthan BR, Moretti L, Cejas D, Singhal D, Hamilton RH, Mohile NA, et al. Advancing Disability Equity in Neurology: An AAN Position Statement. Neurology. 2025;105(3):e213873. doi: 10.1212/WNL.0000000000213873 40601886

[55] Medical Care of Persons with Disabilities H-90.968: American Medical Association; 2022 [July 18, 2025]. https://policysearch.ama-assn.org/policyfinder/detail/Medical%20Care%20of%20Persons%20with%20Disabilities%20H-90.968?uri=%2FAMADoc%2FHOD.xml-0-5283.xml

[56] Position Statement on Discriminatory Disability Insurance Coverage: American Psychiatric Association; 2019 [July 18, 2025]. https://www.psychiatry.org/getattachment/687f96d6-1e12-4fdd-b645-3fca60f2b71c/Position-Discriminatory-Disability-Insurance-Coverage.pdf

[57] American Academy of Pediatrics Council on Children with Disabilities. Care coordination in the medical home: integrating health and related systems of care for children with special health care needs. Pediatrics. 2005;116(5):1238–44. doi: 10.1542/peds.2005-2070 16264016

[58] AMA advocacy efforts: American Medical Association; 2024 [March 19, 2024]. https://www.ama-assn.org/delivering-care/patient-support-advocacy/ama-advocacy-efforts

[59] AMA advocacy efforts: Public health: American Medical Association; 2023 [March 19, 2024]. https://www.ama-assn.org/delivering-care/patient-support-advocacy/ama-advocacy-efforts-public-health#improving-public-health

[60] White Paper Report on Excited Delirium Syndrome: The American College of Emergency Physicians; 2009 [June 18, 2024]. https://www.prisonlegalnews.org/media/publications/acep_report_on_excited_delirium_syndrome_sept_2009.pdf

[61] Saadi A, Naples-Mitchell J, da Silva Bhatia B, Heisler M. End the use of “excited delirium” as a cause of death in police custody. Lancet. 2022;399(10329):1028–30. doi: 10.1016/S0140-6736(22)00410-X 35247310

[62] New AMA policy opposes “excited delirium” diagnosis: The American Medical Association; 2021 [June 18, 2024]. https://www.ama-assn.org/press-center/press-releases/new-ama-policy-opposes-excited-delirium-diagnosis

[63] Position Statement on Concerns About Use of the Term “Excited Delirium” and Appropriate Medical Management in Out-of-Hospital Contexts: The American Psychiatry Association; 2020 [June 18, 2024]. https://www.psychiatry.org/getattachment/7769e617-ee6a-4a89-829f-4fc71d831ce0/Position-Use-of-Term-Excited-Delirium.pdf

[64] Hatten BW, Bonney C, Dunne RB, Hail SL, Ingalsbe GS, Levy MK, et al. ACEP Task Force Report on Hyperactive Delirium with Severe Agitation in Emergency Settings: American College of Emergency Physicians; 2021. [1/17/2024]. https://www.acep.org/siteassets/new-pdfs/education/acep-task-force-report-on-hyperactive-delirium-final.pdf

[65] ACEP Reaffirms Positions on Hyperactive Delirium: The American College of Emergency Physicians; 2023 [June 18, 2024]. https://www.acep.org/news/acep-newsroom-articles/aceps-position-on-hyperactive-delirium

[66] AMA Principles of Medical Ethics: American Medical Association. [September 27, 2023]. https://code-medical-ethics.ama-assn.org/principles

[67] Barnett H, Taylor Z, Booker L, Ricklefs C, Veltri K, Ervin DV, et al. Training Among Noncustodial Health Care Workers Caring for Patients Experiencing Incarceration: A Preliminary Investigation. J Correct Health Care. 2023;29(6):411–20. doi: 10.1089/jchc.22.11.0087 37917880

[68] Trestman RL, Ferguson W, Dickert J. Behind bars: the compelling case for academic health centers partnering with correctional facilities. Acad Med. 2015;90(1):16–9. doi: 10.1097/ACM.0000000000000431 25054416

