# Supplementary material for: Medical associations’ guidance on caring for patients experiencing incarceration in the United States
Source: PLoS One. 2025 Sep 3;20(9):e0330361. doi: 10.1371/journal.pone.0330361 (PMC12407455; doi:10.1371/journal.pone.0330361)
Supplement: S1 Table — This table lists the nine associations excluded from the systematic Internet searches due to specialty irrelevance to carceral healthcare. (DOCX) [file pone.0330361.s001.docx]

**Table S1. Excluded Medical Associations from Analysis**

| **Medical Association Excluded** |
| --- |
| - Aerospace Medical Association - American Academy of Cosmetic Surgery - American Academy of Insurance Medicine - American Contact Dermatitis Society - American Society for Reconstructive Microsurgery - American Society of Cytopathology - International Society of Hair Restoration Surgery - Society for Investigative Dermatology, Inc. - Undersea and Hyperbaric Medical Society |
