# Supplementary material for: Medical associations’ guidance on caring for patients experiencing incarceration in the United States
Source: PLoS One. 2025 Sep 3;20(9):e0330361. doi: 10.1371/journal.pone.0330361 (PMC12407455; doi:10.1371/journal.pone.0330361)
Supplement: S2 Table — Forty-four documents were identified through systematic Internet searches. This table catalogs them by association and summarizes the primary focus of each association’s collection of documents. URLs are provided where available; if a document is no longer publicly accessible, no URL is listed. (DOCX) [file pone.0330361.s002.docx]

**Table S2. Documents Identified by Association**

| **Professional Medical Association** | **Number of Documents Published** | **Primary Focus of Documents** | **Title of Each Document** | **URL** |
| --- | --- | --- | --- | --- |
| AMDA - The Society for Post-Acute and Long-Term Care Medicine | 1 | Education for clinicians on the increasing number of older people who are incarcerated and their impact on the long-term care continuum | Addressing an Expected Increase in Long Term Care Continuum Residents with Criminal/Correctional Histories | No longer publicly available |
| American Academy of Child and Adolescent Psychiatry | 4 | Advocacy for healthcare access, human rights, and dignity | President Letter to ICE | https://www.aacap.org/App_Themes/AACAP/docs/Advocacy/AACAP-Letter-ORR-ICE.pdf |
|  |  |  | Policy Statement on the Jurisdiction of the Juvenile Court System | https://www.aacap.org/aacap/Policy_Statements/2023/Policy_Statement_Policy_Statement_Jurisdictio_Juvenile_Court_System.aspx |
|  |  |  | Solitary Confinement of Juvenile Offenders | https://www.aacap.org/aacap/Policy_Statements/2012/Solitary_Confinement_of_Juvenile_Offenders.aspx |
|  |  |  | Transgender Youth in Juvenile Justice and other Correctional Systems | https://www.aacap.org/aacap/Policy_Statements/2016/Policy_Statement_on_Transgender_Youth_in_Juvenile_Justice_and_other_Correctional_Systems.aspx |
| American Academy of Family Physicians | 3 | Education for clinicians and advocacy for healthcare access and criminal legal system reform | Incarceration and Health: A Family Medicine Perspective (Position Paper) | https://www.aafp.org/about/policies/all/incarceration.html |
|  |  |  | Issues Related to the Incarceration and Detention of Minors | https://www.aafp.org/about/policies/all/incarceration-minors.html#:~:text=The%20AAFP%20maintains%20the%20following,should%20not%20receive%20life%20sentences |
|  |  |  | Restraints on Incarcerated Women During Labor, Use of | https://www.aafp.org/about/policies/all/restraints-on-incarcerated-women-during-labor.html |
| American Academy of Psychiatry and the Law | 2 | Clinical and ethical guidelines for clinicians | Ethics Guidelines for the Practice of Forensic Psychiatry | https://www.aapl.org/ethics.htm |
|  |  |  | The American Academy of Psychiatry and the Law Practice Resource for Prescribing in Corrections | https://www.aapl.org/docs/pdf/Corrections-Resource-Document.pdf |
| American College of Emergency Physicians | 2 | Clinical and logistical guidance on the care of patients who are incarcerated in the emergency department | Recognizing the Needs of Incarcerated Patients in the Emergency Department | https://www.acep.org/administration/resources/recognizing-the-needs-of-incarcerated-patients-in-the-emergency-department |
|  |  |  | ACEP COVID-19 Field Guide: Incarcerated Population | https://www.acep.org/corona/covid-19-field-guide/special-populations/incarcerated-population |
| American College of Obstetricians and Gynecologists | 7 | Advocacy for improved and dignified healthcare for female patients experiencing incarceration | COVID-19 Public Health Considerations for Pregnant and Postpartum People Who Are Incarcerated | https://www.acog.org/clinical-information/policy-and-position-statements/position-statements/2021/covid-19-public-health-considerations-for-pregnant-and-postpartum-people-who-are-incarcerated#:~:text=Given%20the%20convergent%20risks%20of,by%20an%20overall%20decrease%20of |
|  |  |  | Health Care for Incarcerated Women | https://www.acog.org/advocacy/policy-priorities/health-care-for-incarcerated-women |
|  |  |  | Health Care for Immigrants | https://www.acog.org/clinical/clinical-guidance/committee-statement/articles/2023/01/health-care-for-immigrants#:~:text=Conclusion-,The%20American%20College%20of%20Obstetricians%20and%20Gynecologists%20supports%20the%20health,care%2C%20regardless%20of%20immigration%20status. |
|  |  |  | Opposition to Immigration Practices that Are Detrimental to the Well-Being of Women and Children | https://www.acog.org/clinical-information/policy-and-position-statements/statements-of-policy/2018/opposition-to-immigration-practices-that-are-detrimental-to-the-well-being-of-all-individuals |
|  |  |  | Reproductive Health Care for Incarcerated Pregnant, Postpartum, and Nonpregnant Individuals | https://www.acog.org/clinical/clinical-guidance/committee-opinion/articles/2021/07/reproductive-health-care-for-incarcerated-pregnant-postpartum-and-nonpregnant-individuals |
|  |  |  | Sterilization of Women: Ethical Issues and Considerations | https://journals.lww.com/greenjournal/abstract/2017/04000/committee_opinion_no__695__sterilization_of_women_.51.aspx |
|  |  |  | Violence and Racism in the Criminal Legal System: A Women's Health Crisis | https://www.acog.org/clinical-information/policy-and-position-statements/statements-of-policy/2020/violence-and-racism-in-criminal-legal-system-a-womens-health-crisis |
| American Academy of Pediatrics | 2 | Education for clinicians on the care of pediatric patients who are incarcerated and advocacy for improved healthcare and criminal legal policy reform | Advocacy and Collaborative Health Care for Justice-Involved Youth | https://publications.aap.org/pediatrics/article/146/1/e20201755/37020/Advocacy-and-Collaborative-Health-Care-for-Justice?autologincheck=redirected |
|  |  |  | Detention of Immigrant Children | https://publications.aap.org/pediatrics/article/139/5/e20170483/38727/Detention-of-Immigrant-Children |
| American College of Occupational and Environmental Medicine | 1 | Clinical and logistical guidance for safe working environments in correctional settings | Occupational and Environmental Hazards of Correctional Settings | https://journals.lww.com/joem/fulltext/2022/03000/occupational_and_environmental_hazards_of.23.aspx |
| American College of Physicians | 2 | Education for clinicians and advocacy for improved treatment of patients experiencing incarceration | Correctional Medicine - A Public Policy Paper of the American College of Physicians | https://www.acponline.org/sites/default/files/acp-policy-library/policies/correctional_medicine_2001.pdf |
|  |  |  | Health Care During Incarceration: A Policy Position Paper From the American College of Physicians | https://www.acpjournals.org/doi/full/10.7326/M22-2370?rfr_dat=cr_pub++0pubmed&url_ver=Z39.88-2003&rfr_id=ori%3Arid%3Acrossref.org |
| American College of Radiology | 1 | Clinical guidance for imaging patients who are incarcerated | ACR Manual on MR Safety | https://edge.sitecorecloud.io/americancoldf5f-acrorgf92a-productioncb02-3650/media/ACR/Files/Clinical/Radiology-Safety/Manual-on-MR-Safety.pdf |
| American Psychiatric Association | 10 | Advocacy for improved healthcare and conditions for patients experiencing incarceration | The Impact of COVID-19 on Incarcerated Persons with Mental Illness | https://www.psychiatry.org/File%20Library/Psychiatrists/APA-Guidance-COVID-19-Incarcerated-Patients.pdf |
|  |  |  | Position Statement on Psychiatric Services in Adult Correctional Facilities | No longer publicly available |
|  |  |  | Position Statement on Competence Evaluation and Restoration Services and the Interface with Criminal Justice and Mental Health Systems | https://www.psychiatry.org/getattachment/e4854169-07a6-4ab9-8375-27c9a9045b15/Position-Competence-Evaluation-Restoration-Services.pdf |
|  |  |  | Position Statement on Growing Fear over Coronavirus Spread and Mental Health Impact in ICE Detention Centers | https://www.psychiatry.org/getattachment/fad0699c-2ada-468e-b6a1-1398b5b3fbe5/Position-Coronavirus-Spread-ICE-Detention-Centers.pdf |
|  |  |  | Position Statement on Engaging Law Enforcement Personnel and Correctional Staff to Address Mental Health and Racial Inequities in Jails and Prisons | https://www.psychiatry.org/getattachment/f130cbd5-82e9-449a-8631-3bcee9123894/Position-Inequities-Jails-Prisons.pdf |
|  |  |  | Position Statement on Mental Health Needs of Undocumented Immigrants | No longer publicly available |
|  |  |  | Position Statement on Orchiectomy or Treatment with Anti-Androgen Medications as a Condition of Release from Incarceration | https://www.psychiatry.org/getattachment/35b0c2aa-313a-4d53-893d-5c569c7dc286/Position-Orchiectomy-Anti-Androgen-Medication-Incarceration-Release-Condition.pdf |
|  |  |  | Position Statement on Psychiatric Participation in Interrogation of Detainees | https://www.psychiatry.org/getattachment/015a4fab-0de9-4c57-96b3-126c38e40e48/Position-Psychiatric-Participation-in-Interrogation-of-Detainees.pdf |
|  |  |  | Position Statement on Psychiatric Services in Adult Correctional Facilities | https://www.psychiatry.org/getattachment/af0301db-f33c-495a-861c-35036c8c91ae/Position-Psychiatric-Services-in-Adult-Correctional-Facilities.pdf |
|  |  |  | Position Statement on Use of Jails to Hold Persons Without Criminal Charges Who Are Awaiting Civil Psychiatric Hospital Beds | https://www.psychiatry.org/getattachment/a981b29c-a2eb-4ba6-9647-1dcc1fd3e37c/Position-Use-of-Jails-Psychiatric-Bed-Shortage.pdf |
| American Society of Addiction Medicine | 4 | Clinical guidance for addiction treatment and opioid use in correctional facilities | Caring for Patients During the COVID-19 Pandemic: Managing Justice Involved People with Addiction During COVID-19 Pandemic | https://downloads.asam.org/sitefinity-production-blobs/docs/default-source/guidelines/covid-19/6-tf_managing-justice-involved-persons-with-addiction-during-the-covid-19-pandemic_final.pdf?sfvrsn=69ba58c2_2 |
|  |  |  | Public Policy Statement on Treatment of Opioid Use Disorder in Correctional Settings | https://downloads.asam.org/sitefinity-production-blobs/docs/default-source/public-policy-statements/2025-final-pps-on-treatment-of-oud-in-correctional-settings.pdf?sfvrsn=74bc3ae0_1 |
|  |  |  | Public Policy Statement on Access to Medications for Addiction Treatment for Persons Under Community Correctional Control | https://sitefinitystorage.blob.core.windows.net/sitefinity-production-blobs/docs/default-source/advocacy/2021-policy-statement-on-correctional-control_final.pdf?sfvrsn=c3885ac2_3 |
|  |  |  | Access to Medications for Addiction Treatment in Correctional Settings State Brief | https://www.asam.org/docs/default-source/advocacy/mat-in-crmj-settings-final.pdf?sfvrsn=10a559c2_2 |
| Infectious Diseases Society of America | 2 | Clinical guidance and advocacy for improved living conditions during the pandemic and healthcare for infectious diseases | Strengthening the Response to COVID-19 in Correctional Facilities | https://www.idsociety.org/globalassets/idsa/public-health/covid-19/covid-19-in-correctional-facilities.pdf |
|  |  |  | HCV Guidance Updates Recommendations for Screening and Treating Key Populations | https://www.hcvguidelines.org/unique-populations/correctional |
| National Association of Medical Examiners | 1 | Clinical guidance on reporting deaths of people in custody | National Association of Medical Examiners Position Paper: Recommendations for the Definition, Investigation, Postmortem Examination, and Reporting of Deaths in Custody | https://pmc.ncbi.nlm.nih.gov/articles/PMC6474445/ |
| American Medical Association | 1 | Advocacy for better pain management for patients experiencing incarceration | AMA Opioid Task Force issues new recommendations to urge policymakers to protect patients' access to evidence-based treatment, remove barriers to comprehensive pain care | https://end-overdose-epidemic.org/wp-content/uploads/2020/06/2019-AMA-Opioid-Task-Force-Recommendations-FINAL.pdf |
| American Academy of Ophthalmology | 1 | Logistical guidance on billing for the care of patients detained in prisons and jails | How to Bill for Patients Seen While Incarcerated | https://www.aao.org/practice-management/news-detail/how-to-bill-patients-seen-while-incarcerated |
