# Supplementary material for: Medical associations’ guidance on caring for patients experiencing incarceration in the United States
Source: PLoS One. 2025 Sep 3;20(9):e0330361. doi: 10.1371/journal.pone.0330361 (PMC12407455; doi:10.1371/journal.pone.0330361)
Supplement: S3 Table — This table presents example excerpts illustrating each advocacy-oriented theme. (DOCX) [file pone.0330361.s003.docx]

**Table S3. Focus of Advocacy-oriented Policy Statements**

| **Theme** | **Medical Association** | **Title** | **Quote** |
| --- | --- | --- | --- |
| **Policy Content Areas** | | | |
| Correctional Healthcare | **American Society of Addiction Medicine** | **Public Policy Statement on Treatment of Opioid Use Disorder in Correctional Settings** | The American Society of Addiction Medicine recommends: 1. Access to evidence-based [Opioid Use Disorder,] OUD[,] treatment including all FDA-approved medications, either on site or through transport, is the standard of care for all detained or incarcerated persons. |
| Criminal-Legal System | **American Academy of Family Physicians** | **Incarceration and Health: A Family Medicine Perspective (Position Paper)** | Furthermore, as incarceration and detention are themselves detrimental to health, the [American Academy of Family Physicians,] AAFP[,] supports reducing sentences for nonviolent and drug possession offenders and ending detention for those seeking legal asylum in the U.S. |
| Living Conditions | **American Academy of Pediatrics** | **Advocacy and Collaborative Health Care for Justice-Involved Youth** | In 2016, the [American Academy of Pediatrics,] AAP[,] endorsed the United Nations position and the [American Academy of Child and Adolescent Psychiatry,] AACAP[,] policy statement on solitary confinement of juvenile offenders and opposed the use of solitary confinement for juveniles in correctional facilities. |
